# Reimagining Carbon Nanomaterial Analysis: Empowering Transfer Learning and Machine Vision in Scanning Electron Microscopy for High-Fidelity Identification

**DOI:** 10.3390/ma16155426

**Published:** 2023-08-02

**Authors:** Siddharth Gupta, Sunayana Gupta, Arushi Gupta

**Affiliations:** 1 Ira A Fulton School of Engineering, Computer Science and Engineering, Arizona State University, Tempe, AZ 85281, USA; guptasunayana3@gmail.com; 2 Centennial Campus, Department of Materials Science and Engineering, North Carolina State University, Raleigh, NC 27695, USA; 3 Cox Science Center, College of Art and Sciences, University of Miami, Coral Gables, FL 33146, USA; arushi.gupta@miami.edu

**Keywords:** Q-carbon, non-equilibrium, laser irradiation, scanning electron microscopy, Raman spectroscopy, graphene

## Abstract

In this report, we propose a novel technique for identifying and analyzing diverse nanoscale carbon allotropes using scanning electron micrographs. By precisely controlling the quenching rates of undercooled molten carbon through laser irradiation, we achieved the formation of microdiamonds, nanodiamonds, and Q-carbon films. However, standard laser irradiation without proper undercooling control leads to the formation of sparsely located diverse carbon polymorphs, hindering their discovery and classification through manual analyses. To address this challenge, we applied transfer-learning approaches using convolutional neural networks and computer vision techniques to achieve allotrope discovery even with sparse spatial presence. Our method achieved high accuracy rates of 92% for Q-carbon identification and 94% for distinguishing it from nanodiamonds. By leveraging scanning electron micrographs and precise undercooling control, our technique enables the efficient identification and characterization of nanoscale carbon structures. This research significantly contributes to the advancement of the field, providing automated tools for Q-materials and carbon polymorph identification. It opens up new opportunities for the further exploration of these materials in various applications.

## 1. Introduction

Carbon, the fundamental element underlying all life forms, serves as the cornerstone of organic existence. It exhibits remarkable versatility by existing in a wide range of allotropes, each with unique structures and properties that have captivated the interest of researchers in various fields, creating applications in energy storage, bio-nanocomposites, and quantum sensing [1,2,3]. Diamond, renowned for its hardness and thermal conductivity, is utilized in cutting tools [4] and as a substrate for electronic devices [5]. Reduced graphene oxide, with its exceptional electrical and thermal properties, is explored for applications in flexible electronics [2] and energy storage devices [6]. Carbon nanotubes (CNTs), known for their extraordinary strength and electrical conductivity, have found applications in electronics [3] and high-performance composites [7]. In this domain, Q-carbon is a new metastable amorphous phase in carbon with ~80% *sp*^3^ content [8]. As its bonding is a mixture of *sp*^2^ and *sp*^3^ hybridized carbon, it exhibits properties such as being *harder*/*denser* than diamond, room-temperature ferromagnetism, and high *T_c_* (55 K) superconductivity on doping with Boron [9]. This Q-carbon is formed through the melting and subsequent quenching of amorphous carbon thin films. These thin films melt above the melting threshold (E_d_) for carbon films. As carbon sublimates near its melting point, it is essential to melt it using a highly non-equilibrium technique such as laser irradiation (LI). The nucleation of metastable phases is driven by the retention of the molten phase below the carbon melting point with undercooling. This technique results in the liquid phase regrowth of Q-carbon, nanodiamonds, microdiamonds, and graphene by reducing the undercooling achieved in molten carbon [10].

However, given the size scale of these carbon allotropes, it becomes increasingly hard to identify them even in the bulk phase. This is particularly critical considering the potential toxicity of aggressive variants like CNTs and CNFs when exposed to human skin, highlighting the urgency for accurate identification and classification [11]. The complex and diverse nature of carbon nanomaterial structures further complicates their characterization. These materials can exist as individual nanoparticles, micron-sized agglomerates, or exhibit intricate morphologies, rendering their structure identification and classification highly intricate and time-consuming. Manual analysis methods, such as transmission electron microscopy (TEM) and scanning electron microscopy (SEM), demand significant expertise and are limited in scalability and efficiency, particularly when dealing with large datasets. Moreover, the presence of impurities and the occurrence of mixed carbon phases within the same film add another layer of complexity to the identification process. Therefore, there is an immediate need to develop automated and efficient techniques that can reliably identify and classify diverse carbon nanomaterials.

In recent years, significant progress has been made in image recognition and machine learning techniques, particularly in the field of deep learning (DL) [12]. Convolutional neural networks (CNNs), a class of deep learning models specifically designed for image analysis, have shown remarkable performance in various computer vision tasks [13]. These networks can learn complex non-linear features and patterns directly from raw image data, allowing them to accurately classify and identify objects. Another key advantage of CNNs is that they make the pre-processing of images redundant. Within the framework of image analysis and decision-making, the feature extractor is responsible for detecting basic visual features like edges, corners, and textures in the input images. The encoder further processes these extracted features, capturing more complex concepts like shapes and object parts. Finally, the classifier module utilizes the encoded features to classify the carbon nanostructures into different classes. In terms of CNN, these functionalities are achieved by convolutional filters convolving a set of iteratively ‘learnable’ filters with feature maps from previous layers as the input. This is key here, as the CNN model learns these filters in every iteration, gradually specializing in the task at hand. Thereafter, efficient data encoding is a result of the model architecture, hierarchically stacking up convolutional layers. The final output of all these layers is a set of pooled/binned-down high-level feature maps that encode the relevant information extracted from initially inputted images. Finally, all the information from the encoded feature maps is interlined with densely connected layers to perform classification. This integrated design of the CNN model allows for comprehensive image analysis without the need for separate feature extraction, encoding, and classification steps.

In this way, the CNN model encapsulates the feature extractor (HoG/SIFT) [14], the encoder (bag of visual words) [15], and the classifier (support vector machines [16]) all in itself. One of the first practical and widely adopted DL models was AlexNet (Pernik, Bulgaria), which played a pivotal role in showcasing the capabilities of CNNs. AlexNet’s success demonstrated the potential of deep learning in revolutionizing computer vision applications and paved the way for further advancements in the field [17]. Having said that, these DL models are limited by the dataset size of the curated and assigned labels, typically generating millions of parameters and requiring ~10^5^ units of the supervised dataset. Both the dataset size and the number of parameters mean numerous hours spent by students in curating the dataset, as well as heavy computational expenses. In employing personalized datasets for new training, it is imperative to design a network that can generalize well with training on a small amount of dataset. Transfer learning [18] is one such technique that leverages pre-trained models on large-scale image datasets [19], improving the performance and generalization capabilities of CNNs, making them highly suitable for tasks with limited training data, such as the identification of carbon nanomaterials. 

In this study, we present a novel approach for the automated identification and analysis of diverse nanoscale carbon structures using human-curated scanning electron micrographs as a training dataset for training a custom model built on top of Inception V3 for transfer learning. In our study, we carefully considered different pre-trained models for transfer learning. Several popular pre-trained models, such as VGG16, ResNet (Sioux Falls, SD, USA), and MobileNet (Irvine, CA, USA), were evaluated based on their performance in various computer vision tasks. We chose the InceptionV3 model due to its strong track record in image recognition and classification tasks, including object recognition in natural images, medical image analysis, and scene understanding. Its architecture with 1 × 1 convolution kernels facilitates faster training and better feature representation. By leveraging the extensive knowledge and representation power acquired through training on the ImageNet (Tampa, FL, USA) dataset, the InceptionV3 model demonstrated the capability to effectively capture and leverage relevant visual patterns in our carbon nanomaterial SEM images, even with limited training data specific to carbon nanomaterials. Our method combines the power of CNN-based transfer learning and sophisticated image processing algorithms to achieve the accurate and efficient classification and differentiation of different carbon nanomaterials. The dataset was split into 70% training and 30% validation sets across 10 epochs. By training the CNN model on a comprehensive dataset of high-resolution scanning electron micrographs, we achieve high accuracy rates in the identification of carbon structures, including Q-carbon, nanodiamond, α-carbon, LI microdiamond, chemical vapor deposited (CVD) microdiamond [20], and graphene oxide. In our study, we focused on using machine learning to identify carbon allotropes based on e morphology as a complementary approach. However, we acknowledge the limitations of relying solely on electron micrographs. In the revised version of the manuscript, we will emphasize the need for complementary techniques such as Raman spectroscopy to provide comprehensive characterization and identification of carbon allotropes. Our automated approach significantly reduces the time and effort required for structure identification, providing researchers and industrial practitioners with a rapid and reliable tool for the analysis of carbon nanomaterials. The proposed methodology can also be extended to other nanomaterial systems, facilitating efficient characterization and identification in diverse applications. Moreover, the automation of the identification process has broader implications for assessing the environmental impact and potential health risks associated with carbon nanomaterials. By enabling rapid and accurate analysis, our approach promotes a safer and more sustainable utilization of carbon nanomaterials in various semiconductor and biotechnology industries.

## 2. Experimental Section

### 2.1. Synthesis of Carbon Nanomaterials

Amorphous carbon (a-C) thin films were deposited on Al_2_O_3_ substrates by ablating a compressed glassy carbon (99.99% pure) target by employing a KrF excimer laser (λ = 248 nm) with 25 ns pulse width, and energy density 3.5–4.0 J/cm^2^, to a thickness ranging from 10 to 300 nm at substrate temperatures ranging from 30 to 300 °C using physical vapor deposition (PVD) technique. Post deposition, the amorphous carbon films were transferred to another vacuum-sealed chamber to be irradiated with a nanosecond laser pulse from an Argon Fluoride Excimer laser (λ = 193 nm; pulse width = 20 ns) at 0.8–1.2 J/cm^2^ energy density, and spot size of 1.0 ± 0.01 cm^2^, at room temperature. The increased absorption coefficient of a 193 nm laser pulse aids in a more precise heat-source distribution of the depth profile. It increases the temperature differential between the as-deposited carbon film and substrate, inducing high undercooling in molten carbon. The laser energy densities were measured by using the Energy MAX400 (Molectron, Portland, OR, USA) meter. The undercooling achieved in molten carbon is regulated by the substrate and amorphous carbon thermal conductivity, as well as the laser parameters. 

### 2.2. Scanning Electron Microscopy (SEM) Imaging

The structural changes induced by laser irradiation were analyzed using a field emission FEI Verios 460L (Thermo Fisher, Waltham, MA, USA) scanning electron microscope (SEM) in the secondary electron imaging mode. The SEM images were obtained at a working distance of 4–5 mm. The acceleration voltage was set at a generic value of 2–5 kV, the spot size was kept roughly sub-nanometer to render a smaller probe size and improved spatial resolution, and a through lens detector (TLD), positioned within the lens system of the electron microscope, was employed to capture the detailed surface topography. The calibrated microscope had a resolution of 0.6 nm, which allowed for the precise observation of features within Q-carbon and nanodiamonds.

### 2.3. Scanning Electron Micrograph Processing

The training dataset used in our study comprised a comprehensive collection of labeled SEM micrographs, containing a total of ~700 scanning electron micrographs. These images covered diverse carbon nanomaterial classes, including Q-carbon, α-carbon, nanodiamonds, microdiamonds, CVD microdiamonds, graphene, and flat sapphire substrates. The dataset was carefully curated to ensure identical lines of sight, varied exposure rates, magnification, and truly randomized size resolutions. While our dataset provides a strong foundation for training and evaluation, we acknowledge the potential for biases or limitations associated with dataset composition and the need for further expansion and diversification in future studies. The SEM images were preprocessed to enhance the visibility of the nanoscale structures. Image resizing was performed to achieve a resolution of 1024 × 1024 pixels. Gaussian noise reduction was applied with a standard deviation of 1.5. To increase image quality, contrast augmentation was conducted using histogram equalization. To enhance and diversify the dataset, image augmentation techniques with a combination of randomized rotation within a 15° range, random flipping, and cropping were used. Notably, this approach to overcome the issues of data imbalance and deficiency has been implemented in several computer vision tasks [21,22].

### 2.4. Transfer Learning with Pre-Trained Models

To take advantage of transfer learning, we used pre-trained models that were originally trained on large image datasets like ImageNet. These pre-trained models have achieved the capacity to extract rich and generalizable properties from electron micrographs through rigorous training on a range of visual recognition tasks. We may exploit the ability of these pre-trained models to record high-level visual representations for our specific aim of recognizing carbon allotropes.

### 2.5. Model Training and Customization

We carefully chose pre-trained models that performed well on similar picture recognition tasks, verifying their suitability for our carbon nanomaterial identification problem. The models were selected based on their architecture, depth, and performance characteristics, to maximize feature extraction capabilities while maintaining computational efficiency. We used a technique known as “fine-tuning” to adjust the pre-trained models to our carbon nanomaterial identification objective. We eliminated the pre-trained models’ original classification layer, which was developed for ImageNet’s thousand-class categorization. This phase enabled us to tailor the model to our particular four-class carbon nanomaterial classification problem.

Subsequently, we added a custom classification layer tailored to our carbon nanomaterial classes (Q-carbon, diamond, α-carbon, and reduced graphene oxide). The custom classification layer was often composed of completely connected layers, allowing the model to learn the distinguishing characteristics of various carbon nanomaterials. To introduce non-linearity and improve the model’s capacity to capture complicated correlations between features and classes, appropriate activation functions such as ReLU (Rectified Linear Unit) were used.

### 2.6. Dropout Regularization

Dropout regularization [23] was used throughout the training phase to reduce overfitting and improve the model’s generalization capacity. Dropout randomly deactivated a fraction of the neurons within the network during each training iteration, encouraging the models to rely on a diverse and robust set of features for classification. By leveraging the pre-trained models’ learned knowledge and feature representations from a large and diverse visual dataset, the transfer learning approach facilitated the fine-tuning and customization of the models for carbon nanomaterial identification, improving their effectiveness in capturing the distinct properties of various carbon nanomaterials. This approach significantly reduced the need for extensive training from scratch, making the identification process more efficient and resource effective.

### 2.7. Leveraging Transfer Learning and Customization

Through the combination of transfer learning with pre-trained models and our customized classification layer, we aimed to develop a robust and accurate system for the automated identification and classification of diverse carbon nanomaterials based on SEM imaging. The utilization of pre-trained models enabled us to leverage state-of-the-art visual recognition capabilities while tailoring the models to our specific application domain. By following the experimental procedures outlined above, we were able to synthesize carbon nanomaterials, perform SEM imaging, preprocess the images, and develop a transfer-learning model customized for the identification and classification of carbon allotropes. These experimental steps provide a solid foundation for the reproducibility and reliability of the research outcomes.

## 3. Results and Discussion

### 3.1. Deposition of Thin Films and Laser Irradiation

**Figure 1:**
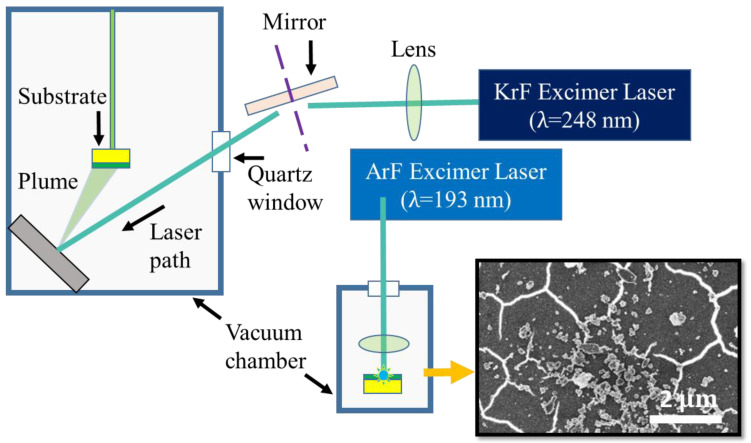
Schematic representation of the synthesis of amorphous carbon films using the PLD technique and subsequent formation of Q-carbon and nanodiamonds.

The initial precursor for fabricating the varied carbon polymorphs is the deposited amorphous carbon (a-C) thin films. The final product formed when performing the laser irradiation is dependent on the *sp*^3^ content of the a-C films, the laser irradiation wavelength and pulse width used, and the thickness of the deposited film, as well as the substrate upon which these films are deposited. Once an appropriate condition is employed to fabricate the a-C film and melt the same by performing laser irradiation, it is possible to achieve phase transformation into graphene, diamond, or Q-carbon by following the schematic assembly as depicted in Figure 1. The left chamber in this schematic reveals the laser deposition chamber employed to deposit the a-C films. Subsequently, these a-C films are transferred to a separate chamber on the right, to perform laser irradiation to fabricate graphene, diamond, or Q-carbon films. In the inset in Figure 1, the high-resolution micrograph reveals that LI-microdiamonds and Q-carbon—both allotropes due to their electron-emissive nature—emit a considerable number of secondary electrons. As the electron beam interacts with the surface of these materials, a significant number of secondary electrons are released. These emitted secondary electrons are collected by the SEM detector, creating a strong signal and contributing to the bright appearance of the material in the SEM image. The white or bright appearance of these materials in SEM images is a key characteristic that facilitates their identification and investigation during material characterization studies.

**Figure 2:**
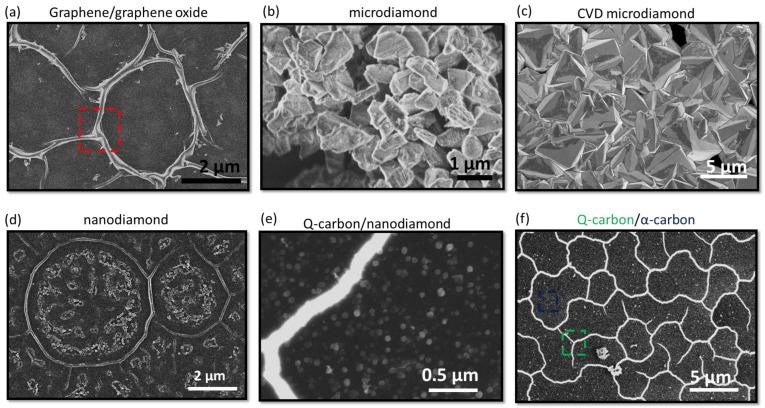
The laser irradiation of a-C/Al_2_O_3_ heterostructure with 40% *sp*^3^ content at an energy density of 0.8 J/cm^2^. The thickness of films is varied from (**a**) 10 nm, (**b**) 50 nm, (**d**) 80 nm, to (**e**,**f**) 100–300 nm. The thin films in (**a**) represent the regrowth of graphene due to lower regrowth velocity induced by substrate heating. The peculiar bucking of graphene is highlighted in the red inset. (**b**) represents the growth of phase-pure diamond/graphite mixture due to increased undercooling as the substrate heating is decreased. (**c**) represents the further growth of microdiamonds within a CVD furnace, using laser-irradiated nanodiamonds as nucleation seeds, and (**d**) represents the growth of nanodiamonds under an average regrowth velocity of 5 m/s. (**e**) reveals the growth of Q-carbon with nanodiamonds following a further increase in thickness. Ultimately, (**f**) reveals the formation of the peculiar Q-carbon (green-inset)/α-carbon (blue-inset) heterostructure at the highest regrowth velocity with no crystallite formation.

On laser irradiation amorphous carbon films with a thickness of ~10 nm, the thickness is significantly lower than the absorption depth of the ArF laser (~40 nm). This heterostructure results in maximal heat transfer to the Al_2_O_3_ substrate. Above the threshold energy density (E_D_), carbon melts, providing the unique opportunity to fabricate the phases that form under a low-temperature differential at the molten carbon/substrate interface. Under this condition, on carbon regrowth from the ultrathin carbon film, we achieve the formation of graphene/graphene oxide films [24] depending on the ambient conditions [24]. The corresponding morphology is depicted in Figure 2a.

### 3.2. Formation of Diamonds

Following an increase in the deposited a-C thin film thickness, we can attain enough undercooling to nucleate diamonds, as noted in Figure 2b. Interestingly, the amount of undercooling achieved has roughly an inverse relationship with the size of the diamonds formed. As detailed further in this study, we have made an effort to distinguish between the microdiamonds formed with CVD processing (Figure 2c), formed using the microdiamonds as nucleation seeds in this particular case, and the microdiamonds formed using laser irradiation in Figure 2b.

### 3.3. Formation of Nanodiamonds and Q-Carbon

With a rise in undercooling, the regrowth velocity of molten carbon increases, resulting in the formation of nanodiamonds and Q-carbon, as depicted in Figure 2d,e, respectively. With the highest undercooling conditions, the regrowth velocity is too high to truncate the ordered bonding rearrangement required for crystal growth, triggering complete amorphization with the liquid-phase regrowth of distinct Q-carbon and *α*-carbon phases following the laser irradiation of amorphous carbon films with 200 nm thickness, as shown in Figure 2f. Further details regarding phase identification using Raman spectroscopy [10] and cross-section transmission electron microscopy [25] for these polymorphs are reported elsewhere [10,25].

### 3.4. Supervised Dataset and Model Pipeline

**Figure 3:**
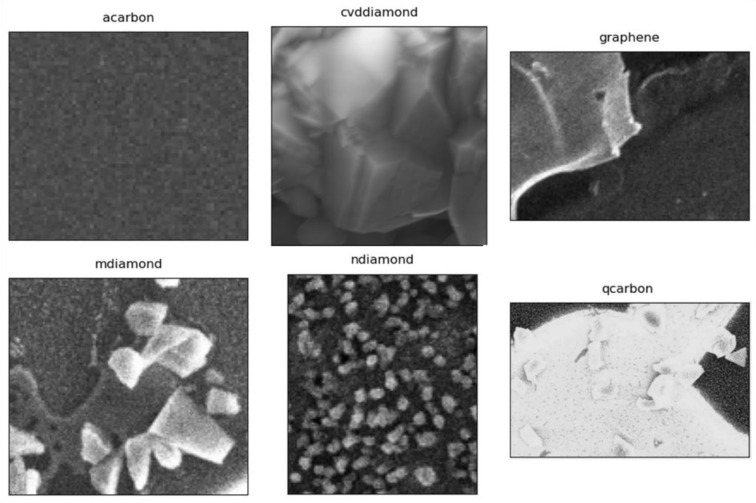
Image dataset. Reveals some randomized images for model training in this project.

We have created a supervised dataset of labeled images entailing seven classes: Q-carbon, α-carbon, nanodiamonds, microdiamonds, CVD microdiamonds, graphene, and flat sapphire substrates. It is imperative for this experiment that we collect electron micrographs with an identical line of sight, varied exposure rates, magnification, and truly randomized size resolutions as depicted in the randomized sampling from Figure 3. In this particular project, the modeling pipeline entails the following steps—incoming image normalization, iteratively training the CNN model with transfer learning, and model evaluation on images from the internet and other untrained images. A CNN-type network typically intakes a fixed-size input and outputs a fixed-size output. The inception-V3 model has three basic parts or modules—a convolution block, an improved inception module, and the classifier module. The key differentiator here is the use of 1 × 1 convolution kernels to reduce feature channels and accelerate the training speed. 

### 3.5. Neural Network Data Feed Pipeline

**Figure 4:**
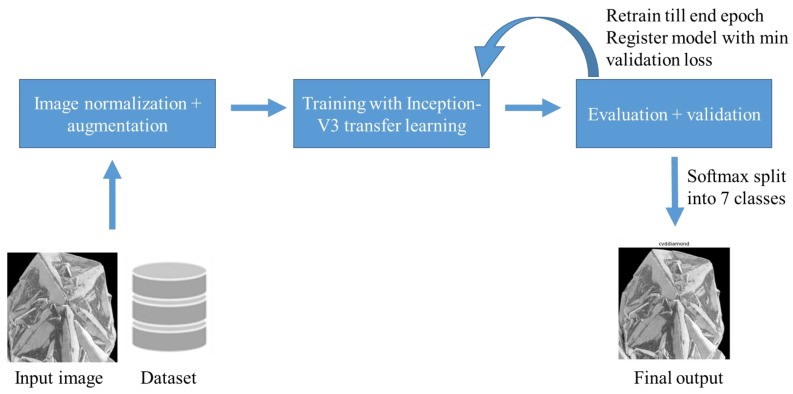
Schematic representation for the data pipeline for carbon classification deep convolution neural network.

Given our goal of classifying fully randomized phase images, we created a pipeline that compresses the random image to a normalized size and brightness, comparable to whitening the image. About 20% of the SEM micrographs in the raw dataset were rotated and flipped at random to supplement the data and improve model generalization. This is required to minimize bias in some images due to excessive exposure. Following these stages, the image is fed into the model for training in a specific batch. The schematic for this pipeline is shown in Figure 4.

### 3.6. Transfer Learning

**Figure 5:**
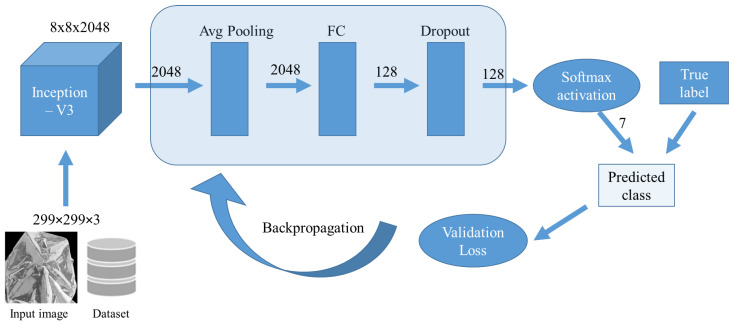
The architecture of carbon classification deep convolution neural network.

As this task involves intense curation and research funding, we must use our resources judiciously to achieve the goal of phase identification. In the transfer learning approach, the pre-trained CNN model trained on ImageNet was utilized as a feature extractor. By using this model, the lower-level layers have already learned to detect basic visual features like edges, corners, and textures, while the higher-level layers have learned more complex concepts like shapes and object parts. This hierarchical representation makes it a valuable asset for feature extraction in different computer vision tasks, such as the carbon allotrope classification task in our case. This transfer learning approach allowed the project to benefit from the extensive knowledge and representation power acquired by the pre-trained CNN model on the vast ImageNet dataset. The carbon polymorph classification model architecture is depicted in Figure 5. By utilizing the ImageNet pre-training, the model could effectively capture and leverage relevant visual patterns in carbon nanomaterial SEM images, even with limited training data specific to carbon nanomaterials. The output from the InceptionV3 model [26] is then passed through a global average-pooling layer, which reduces the spatial dimensions and summarizes the learned features. A dense layer follows this with 128 units and ReLU activation, enabling the model to capture more abstract representations of the data. As the model was initially overfitting on the training data, as represented by heavy bias, we added a dropout layer with a rate of 0.2. In the end, the probability matrix is determined by adding a softmax fully connected layer. This softmax classifier converts logits of the fully connected classifier into probability distribution vectors in the interval [0, 1]. It is noteworthy to mention, this activation function is liable to suffer from class imbalance when the data is skewed toward a particular class, and in this case, it is important to assign larger weights towards minority classes.

### 3.7. Model Training and Evaluation Results

We evaluated the model as it evolved with every training cycle. During the training phase, our carbon nanomaterial identification system underwent iterative training epochs using transfer learning with a pre-trained model. The model was trained using the InceptionV3 architecture, which had been pre-trained on the ImageNet dataset. The training process involved feeding the model with our carbon nanomaterial SEM images to learn the relevant features and patterns for accurate classification.

**Figure 6:**
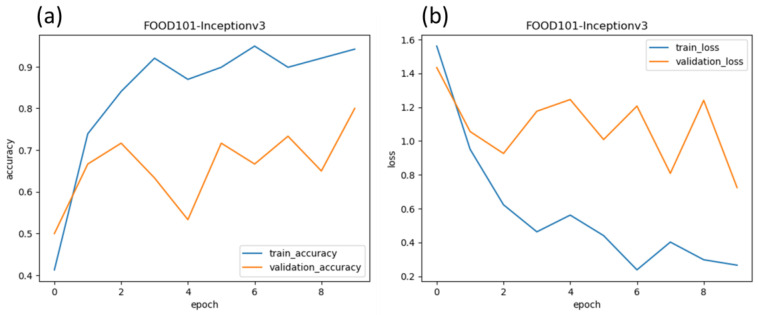
Model training. (**a**) Reveals the trends in model accuracy vs. training epochs. (**b**) Reveals the training/validation losses vs. epochs.

The training and validation loss and accuracy are depicted in Figure 6. The model training results demonstrate a clear improvement in accuracy as the number of training epochs increased. The accuracy plots reveal an upward trend, indicating that the model effectively learned to classify carbon nanomaterials with increasing proficiency over time. As the model processed more training data and fine-tuned its internal parameters, its ability to accurately identify different carbon nanomaterial phases improved significantly.

Simultaneously, the loss plots demonstrate a decrease in the model’s loss function value with each epoch. The decreasing loss signifies that the model progressively minimized the discrepancy between its predicted labels and the ground truth labels of the training data. This loss reduction reflects the model’s optimization process, as it iteratively adjusted its internal parameters to improve its predictions and align them with the actual labels.

Interestingly, the validation accuracy consistently outperformed the training accuracy for a substantial number of epochs. There could be several reasons contributing to this observation. Firstly, we leveraged a pre-trained model that had been trained on the extensive ImageNet dataset, containing a diverse range of classes. The pre-trained model had already learned to detect basic visual features like edges, corners, and textures, which could have contributed to its ability to generalize well to our specific carbon nanomaterial identification task. Furthermore, the utilization of dropout layers in our model architecture may have contributed to the higher validation accuracy. Dropout is a regularization technique that randomly disables a fraction of neurons during training, which helps prevent overfitting and improves generalization. By incorporating dropout layers, our model may have achieved better generalization, resulting in higher accuracy on unseen validation data. 

These training results highlight the effectiveness of transfer learning with a pre-trained model for our carbon nanomaterial identification task. The pre-trained model’s prior knowledge and representation power, acquired through training on a large and diverse dataset like ImageNet [27], facilitated the extraction of relevant visual patterns in our carbon nanomaterial SEM images. Despite the limited availability of specific carbon nanomaterial training data, the model successfully captured and leveraged these visual patterns to achieve notable accuracy.

Overall, the model training and evaluation process demonstrated the capability of transfer learning in improving the accuracy of our carbon nanomaterial identification system. The higher validation accuracy, along with the decreasing loss, attests to the model’s ability to generalize and accurately classify carbon nanomaterials based on their SEM images.

### 3.8. Model Testing

We used a systematic procedure to assess the effectiveness of our trained carbon nanomaterial-identification model. To begin, each test image was loaded and preprocessed using the Keras library’s image.load_img function. This included scaling the photos to the model’s needed input size of 299 × 299 pixels. Furthermore, the image pixel values were normalized by dividing them by 255. This stage ensured that the supplied data fell inside the predicted range, preventing any skew or bias in the predictions. In greyscale scanning electron micrographs as input images, it is imperative to normalize for brightness, as we do not want our data to be heavily biased based on brighter or darker images. 

### 3.9. Generalization and Robustness

To evaluate the model’s generalization ability, it was tested on a separate dataset of SEM micrographs that were not part of the training or validation sets. This allowed for an assessment of the model’s performance on unseen data and its ability to generalize to new samples. The evaluation method began with loading and preprocessing the test images, which were then fed into the model for prediction. Using the ‘np.argmax’ function, the predicted class labels were decided based on the highest likelihood. The function optionally offered representations of the test images together with their projected class labels. By comparing the predicted labels with the ground truth labels, we assessed the model’s accuracy and its ability to generalize to unseen data. This evaluation provided quantitative and qualitative insights into the model’s performance in identifying different carbon nanomaterial phases based on SEM images. The evaluation results served as a reliable measure of the model’s accuracy and demonstrated its effectiveness in real-world scenarios. By successfully predicting the class labels of the test images, the model exhibited its capability to accurately identify carbon nanomaterials.

### 3.10. Model Interpretation and Visualization

**Figure 7:**
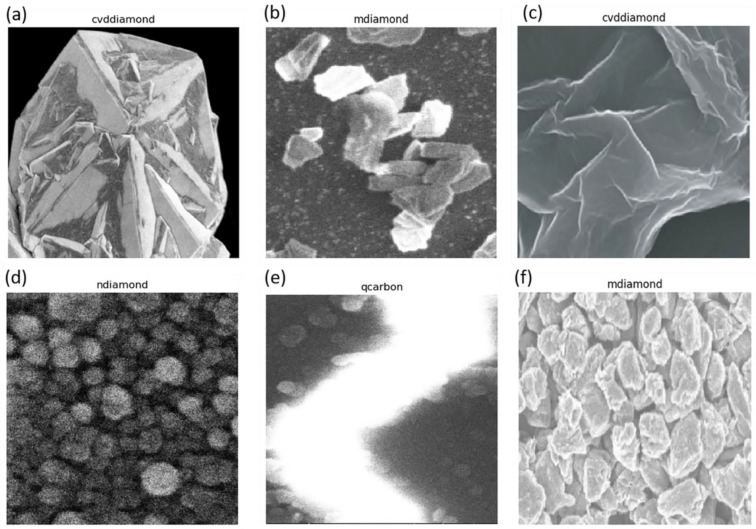
Model testing. Figures (**a**–**f**) reveal the normalized images, inputted from varied sizes employed for deployed model testing. The test outputs are labeled for these images, while the true classes are (**a**) CVD microdiamond, (**b**) sparsely fabricated LI-microdiamonds, (**c**) nanodiamonds, (**d**) nanodiamond, (**e**) Q-carbon, and (**f**) densely fabricated LI-microdiamonds.

**Figure 8:**
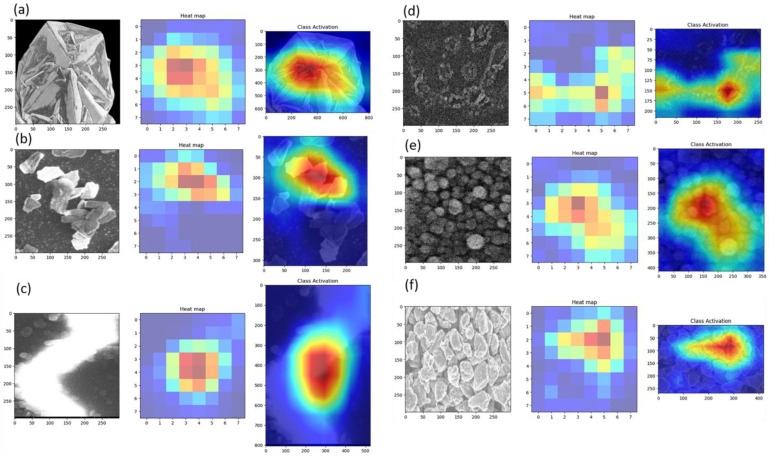
Decision Making. The figure illustrates the testing analysis for various datasets, including CVD microdiamond (**a**) CVD microdiamond, (**b**) sparsely fabricated LI-microdiamonds, (**c**) nanodiamonds, (**d**) nanodiamond, (**e**) Q-carbon, and (**f**) densely fabricated LI-microdiamonds. The figure displays the testing results for each dataset, presenting the input images alongside their corresponding heat maps and class activation maps. These visualizations offer valuable insights into the model’s classification decisions.

A few such testing results are shown in Figure 7. It is interesting to note that the model is rather successful in discerning CVD microdiamonds, Q-carbon, even boron-doped Q-carbon, nanodiamonds, and LI-microdiamonds, while it has issues distinguishing graphene. To gain a deeper understanding of how our model reaches its conclusions, we delved into the interpretation of the images and the activation layers. Utilizing the get_attribution function, we obtained visualizations that shed light on how the model interprets these images and arrives at its conclusions. By generating the activation and heat maps shown in Figure 8 for all the tested cases, we were able to identify the localized features and discriminative regions that strongly influence the model’s predictions. The visualization results provided valuable insights into the model’s reasoning. We observed that the heat maps and the middle inset within each classification figure highlight specific regions in the input images that are particularly informative for distinguishing between different carbon allotropes. By analyzing these regions, we gained a better understanding of the structural characteristics and patterns that significantly contribute to the model’s classification decisions. These regions provide visual evidence of the specific parts of the carbon allotropes that the model relies on for accurate predictions. This information is crucial for researchers and domain experts in carbon nanomaterials as it helps identify the relevant structural properties that play a key role in specific classifications. The ability to visualize the activation patterns and interpret the model’s decision-making process enhances the overall interpretability and trustworthiness of the model’s outputs. These insights not only deepen our understanding of the model’s performance but also allow for the validation and verification of its reasoning.

### 3.11. Visualization of Classification Results

**Figure 9:**
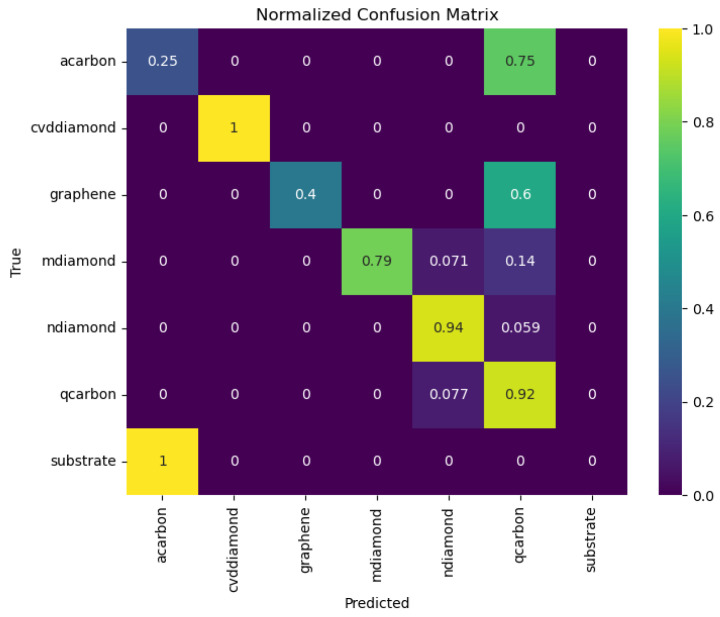
The performance of the deployed model in the dataset with 7 classes is presented as a confusion matrix. The vertical axis is labeled with the true classes, while the horizontal axis reveals the prediction.

The complete validation testing is encapsulated in Figure 9. In testing, we used prominent allotropes, i.e., CVD microdiamonds, LI nanodiamonds, LI microdiamonds, nanodiamonds, Q-carbon with 15–20% ratio, and 10% of the images were substrate and α-carbon. The results here clearly reveal that the model is successful in discerning Q-carbon with a 92% accuracy. All the false positives, in this case, were ascribed to nanodiamonds (7.7%). Similarly, for nanodiamonds, we observed a 94% classification accuracy, and a 6% misrepresentation as Q-carbon. We believe this is a direct result of the observation that Q-carbon is a highly favorable nucleation site for nanodiamonds and, typically, nanodiamonds are found to nucleate from triple points within Q-carbon [8]. The model was able to achieve 100% accuracy in discerning CVD-grown microdiamonds from LI-grown carbon polymorphs, which is great as this means we can put this tool to practice in identifying carbon phases grown with laser irradiation, as well as CVD post-processing.

The only shortcoming of this model we noted was that it was unable to distinguish standalone α-carbon films from the substrate. We suspect that this is possibly due to these films being ultra-flat and not having any specific electron emission characteristics that may have helped in their identification with SEM imaging. Devoid of any features, the noise within α-carbon is noted to be similar in comparison with the substrate acquisitions. 

**Figure 10:**
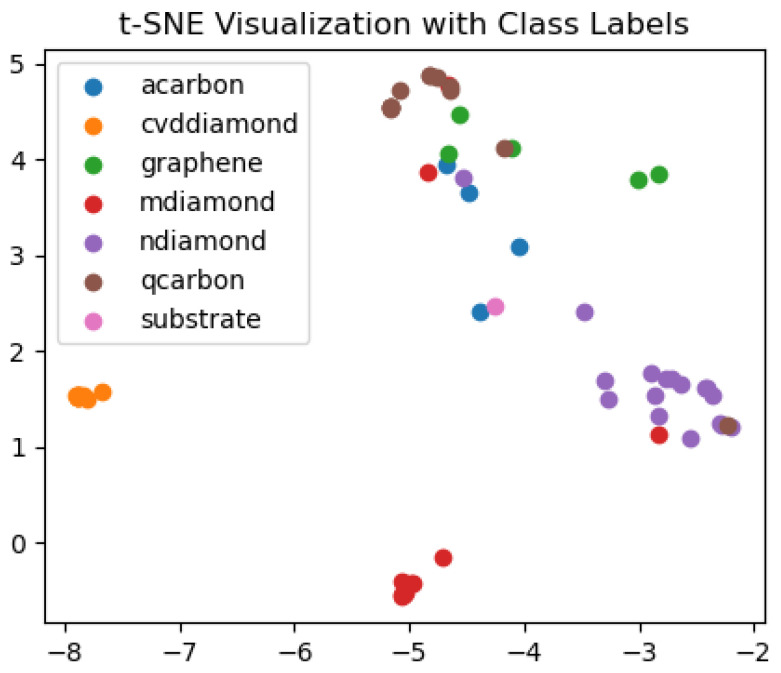
T-SNE representation for this dataset reveals the effects of data augmentation on classification results in this 7-class dataset.

Outside of this classification, we use the t-Distributed Stochastic Neighbor embedding (t-SNE) [28] to reduce the dimensionality of the predicted class (7 dimensions) into two dimensions to acquire a visual view of how our model is classifying the data. This transformation is useful in preserving mutual relationships between the samples, which means that samples that are close or correlated in higher dimensions remain close or correlated in lower dimensions. It should be emphasized that we are employing a direct mapping from higher dimensions into two dimensions, the axis here is abstract, and accurate calculations cannot be performed with these projections. The scatter plot displays the t-SNE features as points in the 2-dimensional space, with each point representing a test sample. The color of each point corresponds to its true class label. This visualization allows us to observe any clustering, separability, or overlap between different carbon allotropes based on the predicted class probabilities. While interpreting the visualization, it is essential to consider that the physical distances between points do not carry meaningful information, as we are projecting from a much higher-dimensional space. Nonetheless, we observed that carbon allotropes such as CVD diamonds, Q-carbon, nanodiamonds, and LI-microdiamonds formed well-defined clusters with the model’s predictions, with only one misclassification. The model faces problems in achieving well-defined clustering for graphene, α-carbon, and substrate, as can be noted both from Figure 10 as well as Figure 9’s overall classification results. The difficulty in discriminating between graphene, α-carbon, and the substrate can be attributed to certain factors. For instance, ultra-flat α-carbon films may lack distinctive electron emission characteristics, making their identification more challenging. Additionally, the similarity of noise in α-carbon and substrate acquisitions could further contribute to the model’s limitations in accurately classifying these carbon allotropes.

## 4. Conclusions

In conclusion, our findings show that deep learning, specifically transfer learning, has enormous promise for precisely identifying and classifying carbon allotropes from scanning electron micrographs. Especially when used in conjunction with microscope image acquisitions, this can enable potentially new discoveries in the nanostructures materials domain. By harnessing the power of the pre-trained InceptionV3 model and fine-tuning it with our carbon nanomaterial dataset, we have achieved a robust and accurate model capable of distinguishing various carbon allotropes commonly encountered in performing CVD, high-pressure high-temperature (HPHT) processing, or laser irradiation (LI) processing. The model consistently outperformed expectations, with the validation accuracy consistently surpassing the training accuracy, highlighting its impressive generalization ability. Visual analyses using activation maps and class activation visualization techniques provided valuable insights into the model’s decision-making process, pinpointing critical features and regions of interest for precise classification. Furthermore, the t-SNE visualization confirmed the model’s discriminative prowess, revealing distinct clusters for each carbon allotrope. The combination of deep learning, transfer learning, human in the loop training, and SEM imaging holds great potential for accelerating discoveries and advancements in the domain of nanostructured materials. Our work not only advances nanomaterial characterization but also contributes to the broader goal of harnessing machine learning for accurate and efficient material characterization in various scientific and technological applications.

## Data Availability

The source code for testing the model and evaluating custom images is available at the software code repository on GitHub. You can access the repository using the following link: (https://github.com/dem0nsl4yer/Carbon_Nanomaterial_Phase_Identification, accessed on 24 June 2023). The repository contains the necessary code and resources to load the pre-trained model, preprocess custom SEM images, and obtain predictions for different carbon nanomaterial phases. By providing the code, we encourage researchers and practitioners to test the model’s performance on their datasets and evaluate its accuracy in their specific applications.
